# Distribution of VGLUT3 in Highly Collateralized Axons from the Rat Dorsal Raphe Nucleus as Revealed by Single-Neuron Reconstructions

**DOI:** 10.1371/journal.pone.0087709

**Published:** 2014-02-04

**Authors:** Dave Gagnon, Martin Parent

**Affiliations:** Centre de recherche de l’Institut universitaire en santé mentale de Québec, Department of Psychiatry and Neuroscience, Faculty of medicine, Université Laval, Quebec City, QC, Canada; Max-Delbrück Center for Molecular Medicine (MDC), Germany

## Abstract

This study aimed at providing the first detailed morphological description, at the single-cell level, of the rat dorsal raphe nucleus neurons, including the distribution of the VGLUT3 protein within their axons. Electrophysiological guidance procedures were used to label dorsal raphe nucleus neurons with biotinylated dextran amine. The somatodendritic and axonal arborization domains of labeled neurons were reconstructed entirely from serial sagittal sections using a computerized image analysis system. Under anaesthesia, dorsal raphe nucleus neurons display highly regular (1.72±0.50 Hz) spontaneous firing patterns. They have a medium size cell body (9.8±1.7 µm) with 2–4 primary dendrites mainly oriented anteroposteriorly. The ascending axons of dorsal raphe nucleus are all highly collateralized and widely distributed (total axonal length up to 18.7 cm), so that they can contact, in various combinations, forebrain structures as diverse as the striatum, the prefrontal cortex and the amygdala. Their morphological features and VGLUT3 content vary significantly according to their target sites. For example, high-resolution confocal analysis of the distribution of VGLUT3 within individually labeled-axons reveals that serotonin axon varicosities displaying VGLUT3 are larger (0.74±0.03 µm) than those devoid of this protein (0.55±0.03 µm). Furthermore, the percentage of axon varicosities that contain VGLUT3 is higher in the striatum (93%) than in the motor cortex (75%), suggesting that a complex trafficking mechanism of the VGLUT3 protein is at play within highly collateralized axons of the dorsal raphe nucleus neurons. Our results provide the first direct evidence that the dorsal raphe nucleus ascending projections are composed of widely distributed neuronal systems, whose capacity to co-release serotonin and glutamate varies from one forebrain locus to the other.

## Introduction

Neurons of the raphe nuclei are involved in multitudinous functions, such as the regulation of sleep-waking cycle, the modulation of pain signals and the pathogenesis of mood disorders. This multifaceted role of raphe neurons is possible because they form a widely distributed neuronal system that reaches virtually all major brain structures, as indicated by previous immunolabeling studies [1]. Originally divided into nine entities [2], the raphe nuclei are actually considered to form a small caudal and a large rostral group having distinct efferent projections [3]–[5]. The caudal group comprises medullary raphe nuclei, which project to the spinal cord whereas the rostral group, scattered along the pons and midbrain, contains the dorsal (DRN, B6 and B7) and median (B8) raphe nuclei, which supply about 85% of the serotonin (5-hydroxytryptamine, 5-HT) forebrain innervation [4].

Retrograde double-labeling experiments have suggested that many raphe neurons are endowed with a markedly collateralized axon [6]–[12]. Bulk injections of anterograde tracers have also revealed that the dorsal raphe efferent projections are widely distributed [13]–[16]. This notion was further extended by antidromic invasion experiments [17]. More recently, single-cell recording and labeling were conducted in the rat DRN, but without providing entire axon reconstructions [18], [19]. Similar approach was also used to gather morphological data on 5-HT neurons of the rat medulla [20].

It has previously been reported that vesicular glutamate transporter 3 (VGLUT3), which is responsible for glutamate vesicular packaging, is expressed in the DRN [21]–[25] and VGLUT3 protein has been visualized in many 5-HT axon varicosities in specific target sites [23], [26]–[30]. This observation, in addition to electrophysiological [31] and optogenetic studies [32] indicate that a proportion of DRN neurons might be able to release glutamate as well as 5-HT in their different target sites. This might play a significant role in neuroadaptative plasticity that take place during development and neurological diseases.

In view of the involvement of DRN neurons in various basic brain functions and brain disease, we thought it worthwhile to investigate, at the single cell level, the trajectory and arborization of their ascending axonal projections as well as the distribution of the VGLUT3 protein within their axons. In order to do so, we combined immunofluorescence with a procedure that allows the injection of very small subsets of electrophysiologically identified neurons and the tracing of single anterogradely-labeled axons arising from the DRN in rats. This research has yielded novel findings that should be taken into account if one hopes to reach a more complete understanding of the anatomical and functional organization of the DRN efferent projections.

## Materials and Methods

### Animals

A total of 15 adult male Sprague-Dawley rats with body weight ranging from 300–450 g were used in the present study. Animal work was performed in accordance to the *Canadian Guide for the Care and Use of Laboratory Animals,* and the Université Laval Institutional Animal Care Committee approved all surgical and animal care procedures (certification #2013-113).

### Stereotaxic Injections

The animals were first anaesthetized with a mixture of ketamine (80 mg/kg) and xylazine (10 mg/kg) before their heads were placed in a stereotaxic apparatus. Two microiontophoretic injections of biotin dextran amine (BDA; Molecular probes, Eugene, Or) were made in the DRN of each rat, with the help of the stereotaxic atlas of Paxinos and Watson [33]. Microiontophoretic labeling was carried out with glass micropipettes (tip diameter 2–3 µm) filled with a solution of potassium acetate (0.5 M) plus 2% BDA 10,000 MW (Invitrogen). These electrodes had impedance ranging between 6–12 MΩ and were also used to monitor the extracellular activity of the neuronal populations encountered during the penetration of the micropipette, including the typical spontaneous rhythmic activity of DRN neurons. These recordings were obtained from 1–3 neurons at each injection site. When the target was reached, the pipette was connected to a high compliance iontophoresis device (NeuroData) and the tracer was injected by passing positive current pulse of 250 nA (1 s on/1 s off) for 25 minutes.

### Tissue Processing for Axonal Reconstructions

After a survival period of 7 days, rats were perfused transcardially with 300 mL of ice-cold sodium phosphate-buffered saline (PBS; 50 mM; pH 7.4), followed by 900 mL of 4% paraformaldehyde (PFA) in 0.1 M sodium phosphate buffer (PB; pH 7.4) and 300 mL of sucrose 10% in PB 0.1 M. After a post-fixation of 24 h in a solution composed of one third PFA 4% and two thirds sucrose 30% diluted in PB, brains were cut along the sagittal plane in 60 µm serial sections using a freezing microtome. Sections were processed for the visualization of BDA according to the avidine-biotine-peroxidase method (ABC, Vector Labs) using nickel intensified 3–3′ diaminobenzidene tetrachloride (NiDAB) as the chromagen. In brief, the sections were incubated overnight at 4°C in a solution containing ABC diluted 1∶60 in 0.1 M PBS, pH 7.4, plus 1% normal rabbit serum and 0.1% triton X-100. They were then rinsed twice in PBS and once in Tris buffer. The bound peroxidase was revealed by incubating the sections in a solution containing 0.05% DAB, 0.3% nickel-ammonium sulfate, and 0.003% hydrogen peroxide in 0.05 M Tris buffer, pH 7.6, for 7–10 minutes at room temperature. The reaction was stopped by two washes in Tris buffer followed by two rinses in PBS. To help identifying structures that harbored labeled axons, sections were counterstained for cytochrome oxidase, according to the histochemical protocol of Wong-Riley [34]. The counterstaining was performed before BDA revelation, and nickel-intensified DAB (dark blue reaction) and unintensified DAB (diffuse brown precipitate) were used to reveal BDA and cytochrome oxidase, respectively. Sections were mounted on gelatin-coated slides, dehydrated in graded alcohols, cleared in toluene, and coverslipped with Permount. Labeled axons were reconstructed in three dimensions by using a light microscope equipped with a motorized stage and an image analysis software (Neurolucida, MicroBrightField, Colchester, VT). Entire and individual axonal reconstructions were obtained from serial sagittal sections, each containing at least one axonal segment. By going from one section to another, we were able to follow and reconstruct individually the injected neurons. The terminal fields of labeled neurons were mapped at lower magnifications to determine their topographic localization.

### Immunofluorescence

Some brain sections were also processed for triple immunofluorescence to characterize the distribution of VGLUT3, VMAT2, SERT and 5-HT in BDA-injected neurons. Briefly, the 60 µm-thick sagittal sections were incubated at room temperature in a blocking solution of PBS 0.1 M containing 2% normal serum and 0.1% Triton X-100 for 30 min and then, in the same blocking solution to which primary antiserum against either 5-HT/VGLUT3, SERT/VGLUT3, 5-HT/SERT or VMAT2/VGLUT3 was added (overnight at 4°C). Then, sections were incubated with corresponding secondary antibodies and with streptavidin Texas Red to reveal the BDA in injected neurons for 2 h at room temperature (see Table 1 for details on antibodies, concentrations and specificity). The VMAT2/VGLUT3/BDA immunostaining was performed on adjacent sections labeled for 5-HT/VGLUT3/BDA in order to assess the 5-HT nature of the BDA-injected axon that could be traced from one section to the other.

**Table 1:** List of antibodies.

Antibody	Company	Catalog #	Dilution	Characterization	Reference	
**Primary antibodies**						
Goat α SERT	Santa Cruz	SC-1458	1∶ 500	Incubation of antibody with blockingpeptide abolished labeling		[64]
Rabbit α 5-HT	Sigma	S5545	1∶ 500	Incubation of antibody with 5-HTsolution abolished labeling		[65]
GP α VGLUT3	Millipore	AB5421	1∶ 1000	Same labeling as with other antibody.Incubation with blocking peptideabolished labeling		[66]
Rabbit α VMAT2	Synaptic System	138 302	1∶ 1000	Single strong band at 55 kDa on Westernblots of brainstem preparation		[67]
**Secondary antibodies**						
Alexa 647 donkey α guinea pig	Jackson	706-605-148	1∶ 200			
Alexa 568 donkey α goat	Invitrogen	A11057	1∶ 200			
Alexa 488 goat α rabbit	Invitrogen	A11008	1∶ 200			
Streptavidin Texas Red	Molecular Probes	S-6370	1∶ 200			
Streptavidin 488	Molecular Probes	S-11223	1∶ 200			

### Confocal Image Analysis

Slides were coverslipped with fluorescence mounting medium (DAKO, Ontario, Canada) and the distribution of immunolabeled proteins within the BDA-filled neurons was analyzed by using a confocal microscope (LSM 700, Zeiss) and an image-analysis software (Imaris, Bitplane). After confocal imaging, sections were reincubated in ABC and NiDAB (as above) to visualize and reconstruct, under a bright field microscope, BDA-injected neurons.

## Results

### General Labeling Features and Somatodendritic Arborization

**Figure 1:**
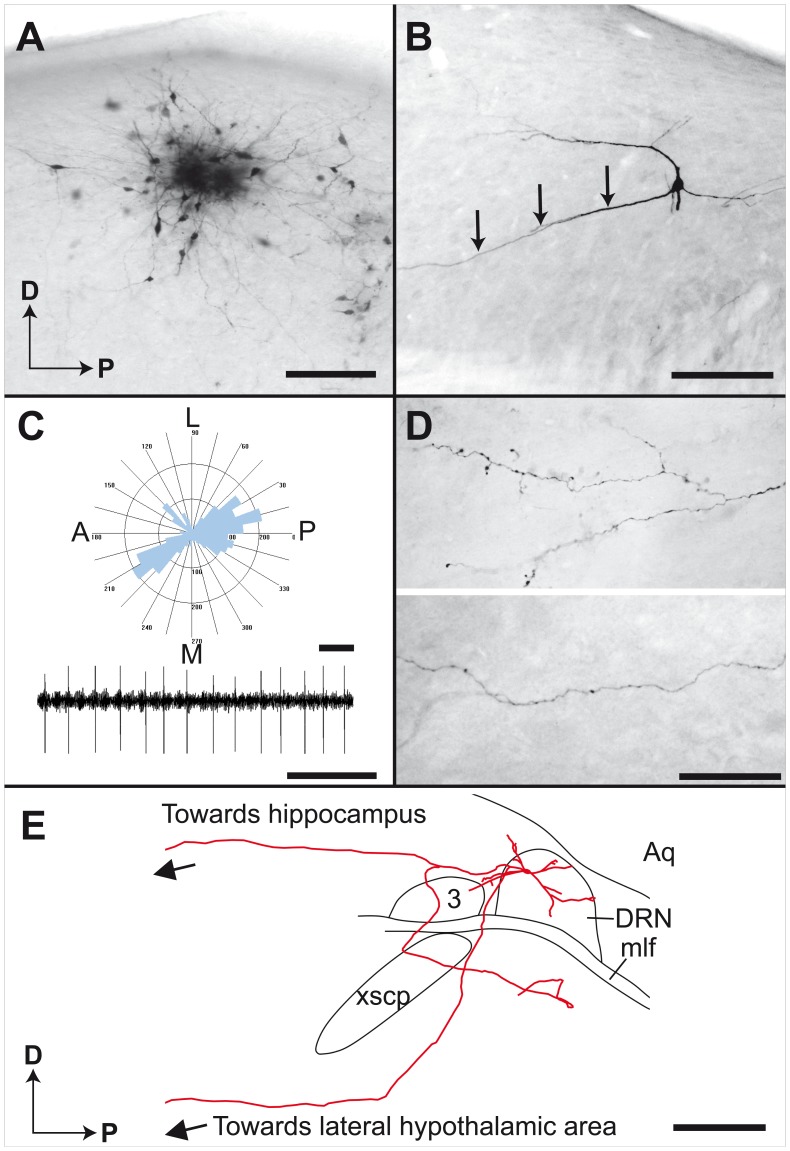
Neurons of the rat DRN filled with BDA. (**A**) Example of an injection site placed in the DRN with a dense core of BDA precipitate and 15 to 20 distinctly labeled neurons. (**B**) Higher magnification of a Golgi-like labeled neuron with 4 primary dendrites. The axon is emitted by a primary dendrite, as indicated by arrows. (**C**) Typical dendrogram of reconstructed neurons showing preferential anteroposterior orientation of the dendrites and patterns of neuronal activity that characterize DRN, as recorded during a single brain penetration with a glass injection micropipette. (**D**) Examples of labeled axons observed in the ventral pallidum (upper panel) and the prefrontal cortex (lower panel). (**E**) Sagittal view of the somatodendritic domain and initial axonal trajectory of a DRN neuron. 3, oculomotor nucleus; A, anterior; Aq, aqueduct; D, dorsal; DRN, dorsal raphe nucleus; L, lateral; M, medial; mlf, medial longitudinal fasciculus; P, posterior; xscp, superior cerebellar peduncle decussation. Scale bars = 150 µm (A), 50 µm (B and D), 200 µm and 2 s (C), 1 mm (E).

The injection procedure used in the present study produces small injection sites involving 15–20 DRN neurons per site. Most injection loci display a dense core of BDA precipitate surrounded by several neurons labeled in a Golgi-like manner (Fig. 1A). The somatodendritic domain (Fig. 1B) and axonal arborization field (Fig. 1D) are entirely labeled. The DRN neurons have a medium sized cell body (9.8±1.7 µm; N = 41) emitting 2–4 long and poorly ramified primary dendrites that are characteristically thick and sparsely spined. Dendrogram analysis reveals that the somatodendritic domain of DRN takes the form of an ellipse (about 700×500×300 µm) preferentially oriented along the anteroposterior axis (Fig. 1C). Such dendritic field often covers the entire anteroposterior axis of the DRN. Dendrites of DRN neurons occasionally extend beyond the boundaries of the DRN (Fig. 1E). Intensely labeled axons emerge from either the core of the injection sites or from individually labeled neurons located peripherally. In the latter case, the axons stems from either the cell body or a primary dendrite (Fig. 1B). Extracellular recordings of BDA-injected neurons in the DRN indicate a highly rhythmic and regular firing pattern with an average frequency of 1.72±0.50 Hz (Fig. 1C).

### Axonal Trajectory

#### General features

The axon of 32 DRN neurons were individually reconstructed in three-dimensions by using a computer image analysis system. Despite a great diversity of axonal branching patterns was noted, many DRN axons follow similar initial trajectories. Most of the reconstructed axons (27/32) pass through the so-called *transtegmental system*, that has been described in details elsewhere [35]. These axons leave the DRN without providing any local collaterals and arch rostroventrally to traverse the central portion of the midbrain tegmentum and reach the decussation of the superior cerebellar peduncle. Only 4 reconstructed axons were seen to travel through the so-called *paraventricular system* [35] by coursing along the dorsal longitudinal fasciculus, en route to the superior and inferior colliculi. These axons then arch ventrally beneath the posterior commissure to reach the lateral hypothalamic area. Interestingly, one DRN neuron had an axon that bifurcate within the confines of the nucleus; one of its branch travels within the transtegmental pathway, while the other courses along the periventricular pathway (Fig. 1E). As they run anteriorly, most labeled axons ascend within the lateral hypothalamic area, along the medial forebrain bundle, except those that innervate caudal structures such as the subthalamic nucleus and the substantia nigra. Labeled axons that course within the lateral hypothalamic area sweep laterally to innervate various components of the forebrain. Along their caudorostral trajectory in the lateral hypothalamic area, axons from the DRN are mostly beaded, but as they reach their target site, their morphological features vary from one locus to the other. In some forebrain structures, axon collaterals are endowed with varicosities “en passant” whereas in others, axons collaterals branch frequently, providing a dense terminal field. It is noteworthy that most DRN neurons provide two types of axonal projections: thin and varicose, and thick and beaded fibers. However, the vast majority of axonal segments are thin and uniform and endowed with fusiform axon varicosities. No contralateral or local axonal projections were observed. Based on the marked variability of axonal branching patterns noted in the different target sites, the population of DRN appears as highly diversified. Representative examples of reconstructed neurons will now be presented according to their major target areas: the striatum, the diencephalon and midbrain tegmentum, the amygdala and septal area and the cerebral cortex. Detailed information on all 32 reconstructed axons is given as supplementary information (Table S1).

#### Striatum

**Figure 2:**
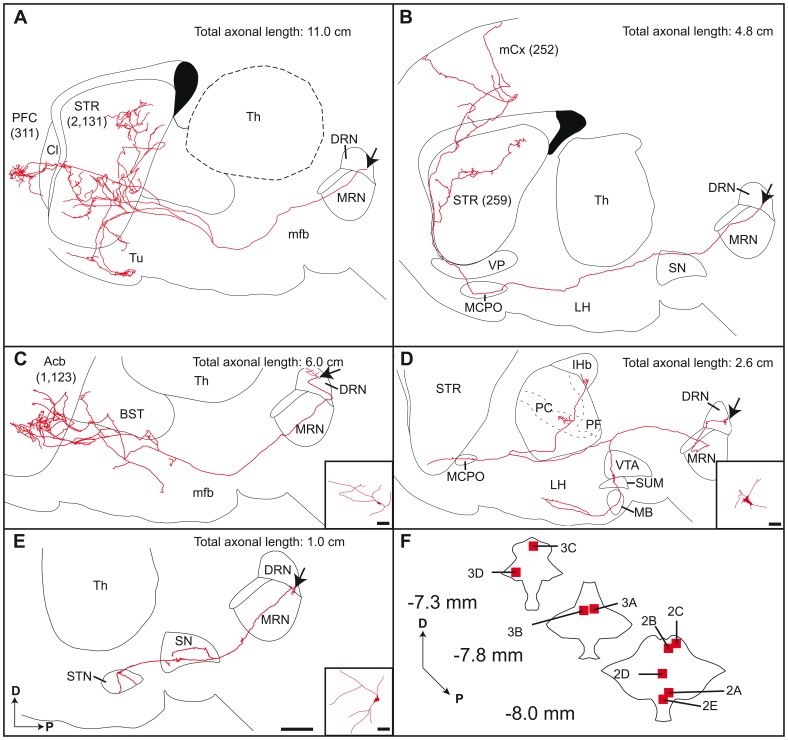
Axonal arborization of DRN neurons, as viewed on sagittal plane. The number of axon varicosities observed in each target site is indicated in parenthesis. Arrows indicate the location of cell bodies. (**A–E**) Composite reconstructions were obtained by superposing all serial sections that contained labeled profiles onto a single two-dimension frame. This way of doing inevitably leads to some image distortion because of the tortuous three-dimension course of the axon and also because the structures in which the axon courses and arborizes are not necessarily at the same plane than the one chosen for the illustration. Hence, the limits of the various structures should be taken as mere indications. This word of caution also applies to figure 3. Inserts in C–E provide reconstructions of somatodendritic domains. (**F**) Schematic representation of 3 rostrocaudal transverse sections through the DRN showing the exact location of parent cell bodies. The numbers refer to panels in which entire axonal arborizations are shown. Acb, accumbens nucleus; BST, bed nucleus of the stria terminalis; Cl, claustrum; D, dorsal; DRN, dorsal raphe nucleus; LH, lateral hypothalamic area; lHb, lateral habenula; MB, mammillary body; MCPO, magnocellular preoptic nucleus; mCx, motor cortex; mfb, medial forebrain bundle; MRN, median raphe nucleus; P, posterior; PC, paracentral thalamic nucleus; PF, parafascicular thalamic nucleus; PFC, prefrontal cortex; SN, substantia nigra; STN, subthalamic nucleus; STR, striatum; SUM, supramammillary nucleus; Th, thalamus; Tu, olfactory tubercle; VP, ventral pallidum; VTA, ventral tegmental area. Scale bar = 1 mm (E, also valid for A–D) and 10 µm (inserts C–E).

Reconstructed neurons that project to the striatum were located rather medially and caudally in the DRN (Fig. 2F and Fig. S2). A striking example of such neuron is illustrated in figure 2A. As for the majority of reconstructed neurons, the main axon exits rostroventrally, enters the medial forebrain bundle and travels through the lateral hypothalamic area. In this area, three main sets of axon collaterals are emitted; they sweep dorsally and remain unbranched until they reach the striatum. There, they break into several short terminal collaterals that spread throughout a large portion of the dorsolateral striatum, considered as the sensorimotor territory. The axon of this particular neuron has a total length of about 11 cm and displays 2,131 axon varicosities in the striatum. Interestingly, the main axonal branch pierces the claustrum to arborize densely in a restricted area of the prefrontal cortex, where it provides only 311 axon varicosities. Two axonal branches bifurcate ventrally to innervate the olfactory tubercle. Another DRN neuron that innervates the striatum (Fig. 2B), emits an axon that passes through the substantia nigra pars compacta, with axon varicosities “en passant”, and courses within the lateral hypothalamic area and the magnocellular pre-optic nucleus. The axon exhibits few varicosities in the ventral pallidum and runs dorsally in the corpus callosum where it emits a major collateral that arborize profusely within a wide area of the sensorimotor territory of the striatum. The main axonal branch enters the motor cortex and divides into thinner collaterals that innervate all cortical layers. The neuron shown in figure 2C has an axon emitting a collateral in the lateral hypothalamic area that runs dorsally towards the bed nucleus of the stria terminalis to arborize profusely in the nucleus accumbens. The main axonal branch ends its course in the nucleus accumbens, where it displays a wide and dense terminal arborization. Few beaded axon collaterals are also observed in the lateral hypothalamic area.

#### Diencephalon and midbrain tegmentum

The axonal arborization of a typical DRN neuron innervating the thalamus is shown in figure 2D. The cell body of this neuron lies centrally in the caudal portion of the DRN (Fig. 2F). The axon runs within the median raphe nucleus before traveling through the paraventricular system where it divides into two main branches, one that sweeps ventrally to innervate the ventral tegmental area, the supramammillary nucleus, the mammillary body and the lateral hypothalamic area, and the other that courses rostrally and dorsally to arborize in the paracentral, parafascicular and lateral habenular nucleus of the thalamus. The main axonal branch terminates within the nucleus accumbens. The neuron depicted in figure 2E emits an axon that arches rostroventrally to travel within the median raphe nucleus and arborize in both the substantia nigra pars compacta and the subthalamic nucleus, two important components of the basal ganglia.

#### Amygdala

**Figure 3:**
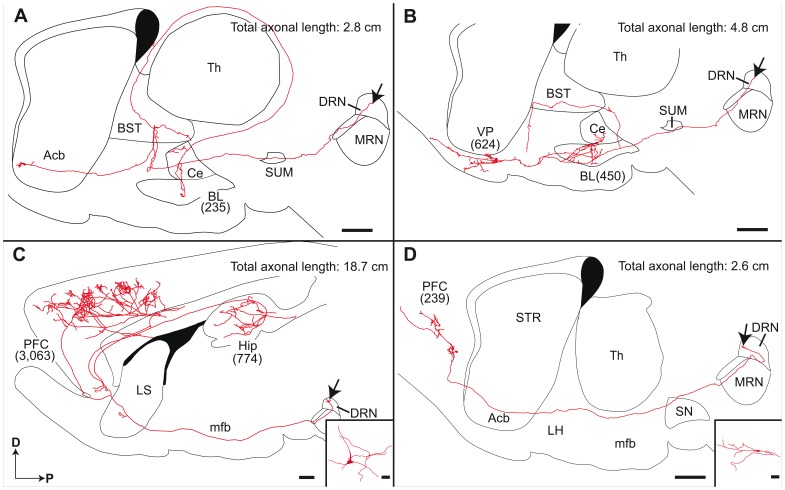
Sagittal view of entire reconstructed axonal arborization of DRN neurons. The number of axon varicosities observed in each target site is indicated in parenthesis. Arrows indicate the location of the parent cell bodies. Inserts in C and D provide recontructions of somatodendritic domains. Acb, accumbens nucleus; BL, basolateral amygdaloid nucleus; BST, bed nucleus of the stria terminalis; Ce, central amygdaloid nucleus; D, dorsal; DRN, dorsal raphe nucleus; Hip, hippocampus; LH, lateral hypothalamic area; LS, lateral septum; mfb, medial forebrain bundle; MRN, median raphe nucleus; P, posterior; PFC, prefrontal cortex; SN, substantia nigra; SUM, supramammillary nucleus; Th, thalamus; VP, ventral pallidum. Scale bars = 1 mm (A–D) and 10 µm (inserts C, D).

Reconstructed neurons that arborize profusely in the amygdala were principally located in the dorsal and caudal portions of the DRN (Fig. 2F and Fig. S2). One of these neurons is depicted in figure 3A. Its axon runs through the supramammillary nucleus and the lateral hypothalamic area, where it gives off a major collateral that ascends dorsally, enters the bed nucleus of the stria terminalis and invades the stria terminalis itself. After a typical loop along the lateral border of the thalamus, this axon collateral sweep laterally to provide a terminal arborization to the central and basolateral nuclei of the amygdala. The main axonal branch terminates in the nucleus accumbens, considered as the limbic territory of the striatum. Another neuron that aims to the amygdala is depicted in figure 3B; its axon innervates profusely the central and basolateral amygdaloid nuclei, as well as the bed nucleus of the stria terminalis and the ventral pallidum.

#### Prefrontal cortex

Neurons that innervate the prefrontal cortex have their cell bodies widely distributed in the DRN (Fig. 2F and Fig. S2), and two neurons of this type are depicted in figure 3 (Fig. 3C, D). Their axon typically runs through the transtegmental system. They ascend through the ventral tegmental area and the lateral hypothalamic area to enter the lateral septum (Fig. 3C) or the nucleus accumbens (Fig. 3D), where they provide a small number of axon varicosities. The axon of both neurons reaches the prefrontal cortex where they arborize within all six cortical layers, providing respectively 3,063 and 239 axon varicosities. The axon illustrated in figure 3C also yields a collateral that enters the subiculum and arborizes profusely in CA1, but also in CA2, CA3 and dentate gyrus.

### Distribution of VGLUT3, VMAT2, 5-HT and SERT within the BDA-filled Axons

**Figure 4:**
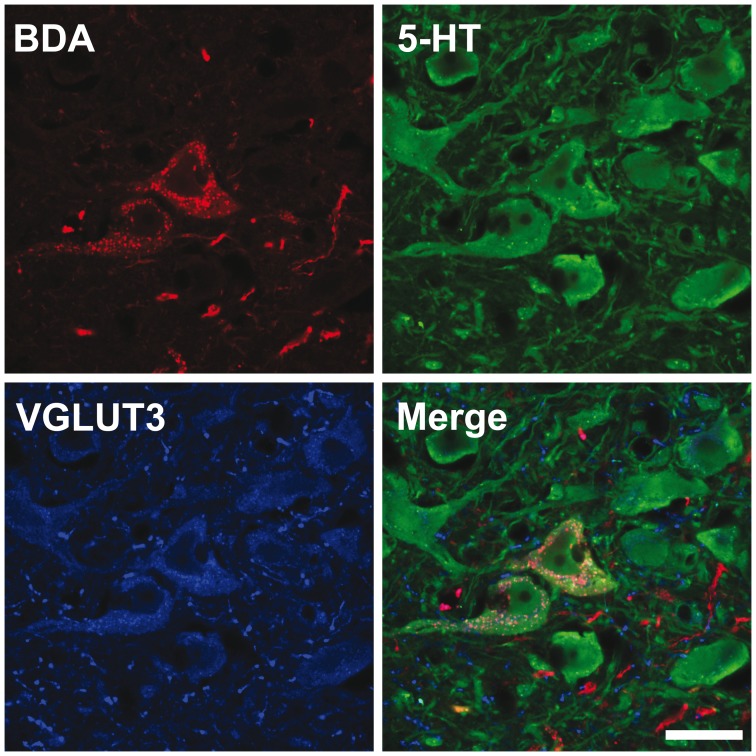
Confocal image of two BDA-injected neurons (red) immunoreactive for 5-HT (green) and VGLUT3 (blue). Scale bar = 10 µm.

**Figure 5:**
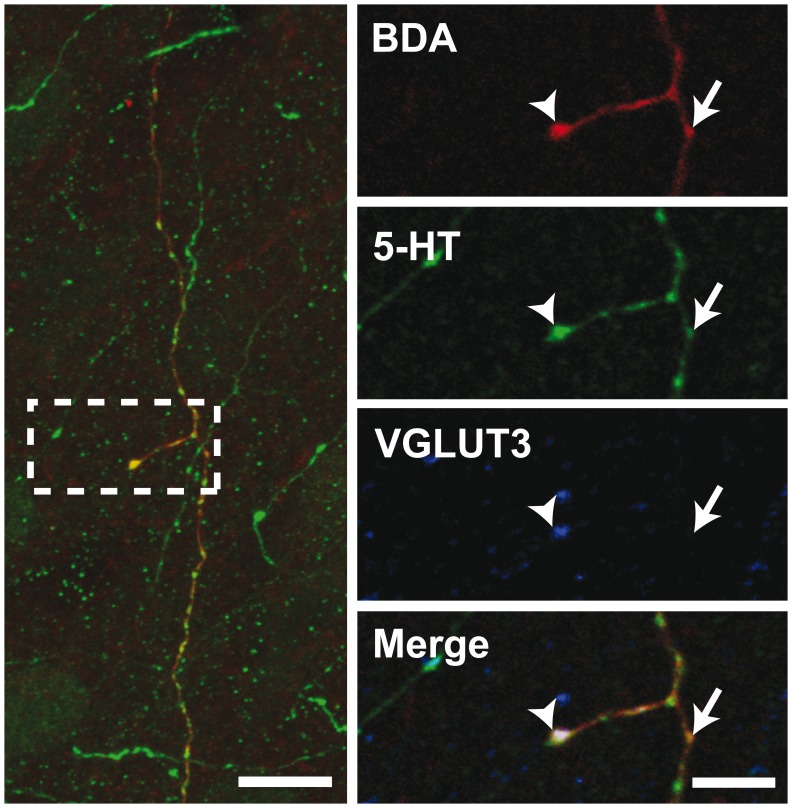
Confocal image of an axonal segment in the rat motor cortex emitted by a DRN neuron injected with BDA. Immunoreactivity for BDA, 5-HT and VGLUT3 are shown in red, green and blue, respectively. Arrowheads show an axon varicosity containing 5-HT and VGLUT3 whereas arrows point to a 5-HT axon terminal devoid of VGLUT3. The left panel is from a 20 µm-thick Z-stack whereas the right panels are single plane images. Scale bars = 10 µm (left) and 5 µm (right).

**Figure 6:**
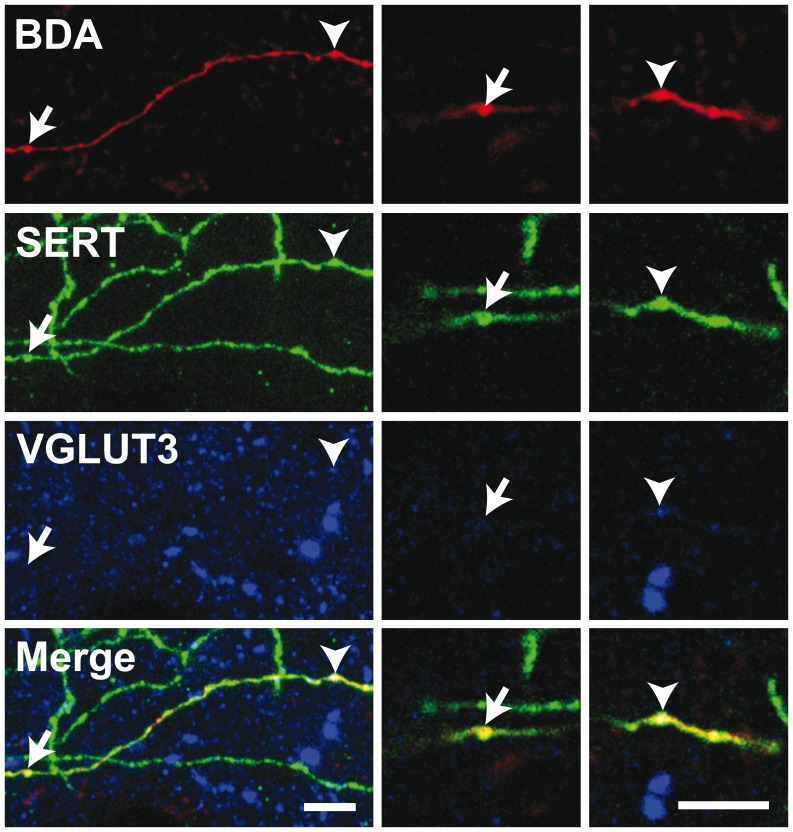
Confocal images of a BDA-filled axonal segment observed in the motor cortex. Immunoreactivity for BDA, SERT and VGLUT3 are shown in red, green and blue, respectively. Arrows indicate an axon varicosity from the BDA-labeled axon that is devoid of VGLUT3 whereas arrowheads point to an axon terminal immunoreactive for SERT and VGLUT3. Left panels are from a 12 µm-thick Z-stack whereas right panels are images from single planes. Scale bars = 5 µm.

**Figure 7:**
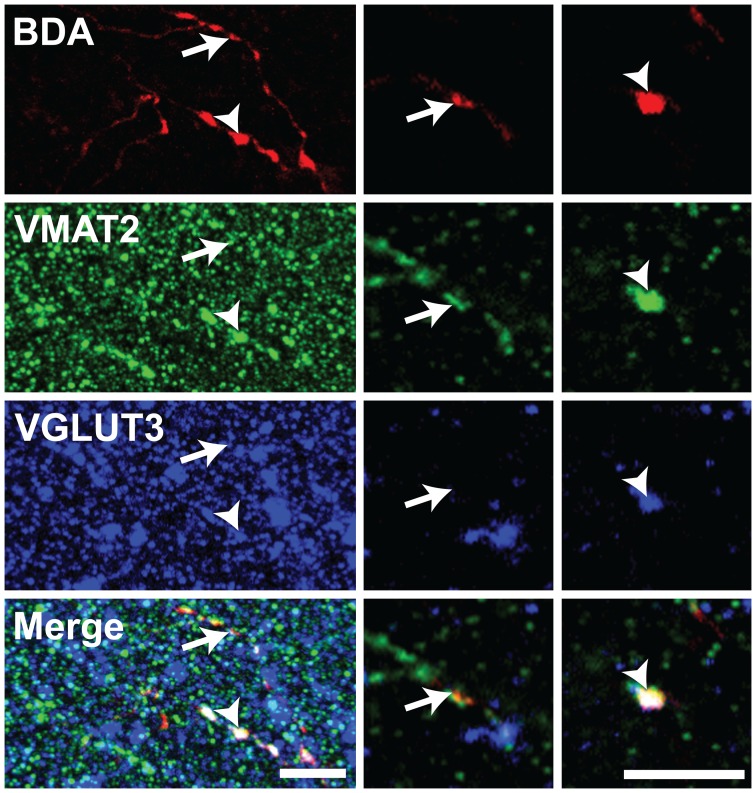
Confocal image of an axonal segment coursing in the motor cortex emitted by a DRN neuron injected with BDA. Immunoreactivity for BDA, VMAT2 and VGLUT3 are shown in red, green and blue, respectively. Arrows point to a BDA-labeled axon varicosity immunoreactive for VMAT2 but devoid of VGLUT3 whereas arrowheads indicate a bouton that contains VMAT2 and VGLUT3. Left panels are from a 30 µm-thick Z-stack whereas right panels are images from single planes. Scale bars = 5 µm.

The 5-HT nature of individually traced axonal segments from the DRN was examined only in the set of experiments designed to investigate the axonal distribution of VGLUT3 at the single-cell level with confocal microscopy. From 156 BDA-labeled cell bodies mainly located in the central portion of the DRN, 117 (75%) were found to display immunostaining for 5-HT. All BDA/5-HT labeled neurons were immunoreactive for VGLUT3 (Fig. 4). All BDA-filled axonal segments and axon varicosities that were observed and traced in the striatum and the prefrontal cortex were immunoreactive for SERT and 5-HT (Fig. S1). Among those varicosities, the vast majority was immunoreactive for VGLUT3. Overall, from 259 axon varicosities located in the motor cortex that were examined in details with the confocal microscope and that belong to 29 distinct axonal segments of BDA-injected neurons (Figs. 5, 6), 75% (193/259) were immunoreactive for VGLUT3, but this percentage reaches 93% (70/75) in the dorsal striatum after the reconstruction of 10 axonal segments. Although the proportion varies depending on the target site, some VGLUT3+ and VGLUT3- axon varicosities were observed along the same BDA-filled axonal segment. 13/29 axonal segments observed in motor cortex and 6/10 in dorsal striatum contained axon varicosities that were all VGLUT3+ whereas no axonal segment were completely devoid of VGLUT3+ boutons. Axon varicosities that contain VGLUT3 were larger than those devoid of this marker (0.74±0.03 µm vs. 0.55±0.03 µm, *P*<0.001). While all BDA-filled axon varicosities and intervaricose axonal segments were immunoreactive for 5-HT, the VGLUT3 protein was restricted to some axon varicosities and absent from intervaricose segments. The vesicular monoamine transporter type 2 (VMAT2) was mostly restricted to axon varicosities but could be seen occasionally in intervaricose segments of BDA-labeled axons. In contrast to the VGLUT3 protein, all BDA-filled axon varicosities that were examined in the prefrontal cortex and the striatum were immunoreactive for the VMAT2 (Fig. 7).

## Discussion

This study has unveiled novel aspects of the organization of DRN ascending projection in the adult rat. By providing detailed reconstructions of single axonal arborization, our work has provided a firm ground for the highly collateralized nature of the DRN axonal projections. Our confocal immunofluorescence analysis has also provided the first demonstration of the precise distribution of VGLUT3, VMAT2, 5-HT and SERT in singly-labeled DRN neurons. Our data indicate that a subset of 5-HT axon varicosities are devoid of the VGLUT3 protein and that this proportion varies depending on target sites. This observation supports the existence of a complex trafficking mechanism of the different types of synaptic vesicles within the highly collateralized axons of the DRN neurons. The present study has also revealed that, based on the diversity of the pattern of their axonal projections, neurons of the DRN form a highly heterogeneous population.

### Organization of the Somatodendritic Domain as an Indication of Integration Capacity

The morphological analysis of the dendritic arborization of DRN neurons have revealed an ellipsoidal shape, measuring approximately 700 µm×500 µm×300 µm, with the longest axis being largely parallel to anteroposterior axis of the nucleus. Such elongated unit may cover the whole rostrocaudal extent of the DRN indicating that a single neuron is able to receive and integrate most of the DRN afferent projections. This columnar arrangement of DRN neurons is in accordance with previously published descriptions [19], [36], [37], including the seminal paper of Cajal [38].

### A highly Collateralized Axon as the Morphological Substratum of Functional Diversity

In accordance to previous study involving bulk injections of anterograde [13]–[16] and retrograde tracers [6]–[9], our data provide direct evidence for the fact that DRN neurons are endowed with a highly collateralized axon that might represent the morphological substratum of the diverse functions played by the 5-HT system. By sending a copy of its efferent message to several target sites, DRN neurons are able to modulate and to possibly synchronize the activity of several functionally diverse brain areas.

Many reconstructed neurons innervate the prefrontal cortex, which is reportedly linked reciprocally to the DRN [39], [40]. Single DRN neurons were observed to innervate both the prefrontal cortex and the hippocampus. It has already been shown that some DRN neurons fire in a time-locked manner to the hippocampus theta rhythm [18], [41]. Our results indicate that the prefrontal cortex activity, which is correlated with hippocampal theta rhythm during spatial working memory task [42], [43], can potentially be modulated by single DRN neuron that innervates both structures.

Some reconstructed DRN neurons, as exemplified in figure 2B, innervate both the substantia nigra and the striatum, a pattern of axonal arborization that allows a single DRN neuron to exert a dual influence upon nigrostriatal dopaminergic neurons. A single DRN neuron can thus act locally at the somatodendritic domain of nigrostriatal dopaminergic neurons, as well as distally by modulating pre-synaptically the dopaminergic axon terminals at the striatal level. The same logic can be applied to the control mesolimbic dopaminergic projection neurons, with single DRN neuron innervating locally the ventral tegmental area and distally the nucleus accumbens (Fig. 2D). Although the neurochemical content of entirely reconstructed DRN neurons was not assessed in the present study, we hypothesize that such a modulation of dopamine by DRN neurons could be mediated through the activation of the 5-HT_2C_ receptors, which occurs at striatal axon terminal levels as well as on the cell bodies of nigral and ventral tegmental dopaminergic neurons [44].

### A Broad Axon Terminal Domain to Influence Wide Neuronal Populations

Our single cell labeling procedure has revealed that DRN neurons target many areas involved in a brain functions that range from the control of motor behaviour to that of limbic functions. Two types of neurons could be identified based on target sites of their axonal arborization: a) those that ramify principally within structures that typically belong to the limbic system, and b) others that branch mostly within brain nuclei associated with motor system. Both types of neurons possess a highly collateralized and widely distributed axon, which is ideally suited for the ubiquitous modulatory role that 5-HT neurons are known to exert at the forebrain level. Our data reveal that the axonal arborization of DRN neurons varies significantly according to their target sites. For example, single-labeled neurons that aim at the prefrontal cortex display a profuse axonal arborization that encompasses all six cortical layers and covers a wide area of cortical tissue. Likewise, striatal afferent axons branch extensively within a large portion of the structure. In contrast, axons that provide terminal branches within the lateral hypothalamic area are very poorly arborized. These findings indicate that the type of axonal branching pattern is not an intrinsic property of DRN neurons, but instead appears to be dependent on the molecular cues that are contained in each terminal site during development [45].

Bulk injections of anterograde tracers combined with immunohistochemistry has revealed the existence of two morphologically distinct types of 5-HT axons in the rat cerebral cortex [46]. Axonal projections from the median raphe nucleus were reportedly enriched with large and spherical axon varicosities, while displaying significant variations in axonal diameter, whereas DRN projections displayed smaller axon diameter with smaller pleomorphic axon varicosities. Our own detailed neuronal reconstructions reveal that single labeled DRN neurons can display both of these types of axons in the cerebral cortex. However, in accordance with Kosofsky and Molliver’s descriptions, the vast majority of DRN axonal segments disclosed on our material are of small and uniform diameter and endowed with fusiform axon varicosities.

As they emerge the DRN, axons bear a significant number of varicosities that appear to establish contact “en passant”. Such a phenomenon also occurred in white matter tracks such as the corpus callosum or the median lemniscus. Electron microscopic studies have shown that, in many brain areas, a significant proportion of 5-HT axon varicosities are devoid of synaptic contacts. Such is the case in the rat cerebral cortex [47] and subthalamic nucleus [48], where approximately half of 5-HT axon varicosities were asynaptic [49]. This feature has been viewed as morphological evidence for the existence of diffuse transmission by 5-HT systems, in addition to their synaptic mode of transmission [50]. It has also led to the suggestion that a low, ambient level of 5-HT might permanently exist in the extracellular space, the fluctuations of which could regulate a variety of physiological processes mediated by 5-HT and its receptors widely distributed on neuronal, glial and vascular elements. The partially synaptic character of 5-HT system combined to highly collateralized axons providing many axon varicosities “en passant” support the modulatory nature of 5-HT. Convincing electrophysiological evidence for a regulatory role of ambient 5-HT has already been obtained in the rat substantia nigra [51].

### Number of Axon Varicosities as an Indication of Input Strength

While reconstructing DRN neuron individually, we paid a particular attention to the number of axon varicosities emitted in the different terminal fields, as it provides an indication of the input strength of single DRN neurons in the various forebrain nuclei. It also allows to approximate the amount of neurotransmitter released by single DRN neuron since the number of axon varicosities has been shown to be correlated with extracellular neurotransmitter concentrations [52]. Neurons that innervate the prefrontal cortex display a high degree of variability in terms of the number of axon varicosities, which ranges from 85 to 3,063. It has been proposed that a given neuron is limited in its total length, as well as in the number of axonal branch and varicosities that it can emit [53]. This hypothesis can be illustrated by comparing the axonal arborization of the neuron shown in figure 3C, that provides 3,063 varicosities in the PFC and only 774 in the hippocampus, to the one illustrated in figure 2A that provides only 311 boutons in the PFC, but 2,131 in the striatum. Again, single-neuron reconstructions in the DRN suggest that a given neuron is limited in the number of projections that it can provide. A high degree of axon collateralization might allow exquisitely precise interactions between various brain structures but the maintenance of this morphological feature implies high-energy consumption, which might represent a limiting factor in the extent of axonal aborization that a given neuron can provide [53].

### VGLUT3 Content of 5-HT Axon Varicosities as a Factor that Favors Neuroplasticity

It has recently been shown that some 5-HT cell bodies located in the DRN express *VGLUT3* [22]–[25], [54], [55] and that only a subset of axon varicosities are immunoreactive for the protein, as indicated by the present immunofluorescence analysis of reconstructed neurons and by previous immunohistochemical studies [26], [27], [29], [30], [56]. Evidence of co-release of glutamate and 5-HT by DRN neurons has been gathered using electrophysiology [31] and optogenetic [32]. Other *in vitro* experiments have indicated that VGLUT3 positively modulates 5-HT transmission [26], probably through a mechanism termed vesicular-filling synergy in which glutamate co-entry in synaptic vesicles promotes storage of 5-HT by increasing the pH gradient that drives VMAT2 [57], [58]. Our triple immunofluorescence confocal investigation clearly shows that all axon varicosities from BDA-injected neurons of the DRN contain VMAT2, SERT and 5-HT. This finding is at odd with a previous report that indicates very sparse colocalization of SERT and 5-HT immunolabeling in mice [26]. Whether this discrepancy reflects a methodological variant or a genuine interspecies difference remains to be determined.

In agreement with previous reports [26], [27], [29], [30], [56], our results indicate that some 5-HT axon varicosities are devoid of VGLUT3. Furthermore, we detected the presence of both VGLUT3+ and VGLUT3- axons varicosities in all target sites investigated, and these two types of varicosities often occurred along the same 5-HT axonal segment. However, we found that the proportion of axon varicosities that contain VGLUT3 is target-site dependent, a finding that supports the hypothesis of a trafficking mechanism of the VGLUT3 protein within the highly collateralized axons of the DRN neurons. We hypothesise that VGLUT3 proteins are located on the same synaptic vesicles than the VMAT2, as it appears to be the case for vesicular acetylcholine transporter (VAChT) and VGLUT3 in cholinergic axon varicosities of the striatum [57] and for VGLUT2 and VMAT2 in dopaminergic striatal terminals [58]. However, our data clearly indicate the existence of a pool of 5-HT synaptic vesicles that contain VMAT2 without VGLUT3, since many VMAT2+/VGLUT3- axon varicosities were observed.

The putative capability of a given DRN neuron to release 5-HT and glutamate together or 5-HT alone is highly relevant for plasticity and neuroadaptative mechanisms that are crucial during development, aging and pathological process. As mentioned above, electron microscopic study has revealed that the synaptic incidence of 5-HT axon varicosities varies depending on target sites but is rather low compared to the glutamatergic system that appears to be entirely synaptic [59], and this has been viewed as the morphological substrate for volume transmission of 5-HT. Moreover, because the existence of junctional complexes implied some structural stability, the converse was suggestive of some mobility of the releasing sites. This has already been considered as a determinant of the remarkable capacities of 5-HT neurons for regeneration in contrast to more hardwired systems [60], [61]. It is tempting to speculate that axon varicosities that are devoid of VGLUT3 would show less synaptic contact than those that contain both transmitters. Along this line, it has been reported that dopaminergic axon varicosities that contain VGLUT2 are more synaptic than those that do not [59], [62]. It is also noteworthy that 5-HT axon varicosities that contain the VGLUT3 were larger, in congruence with the previously reported data that larger 5-HT axon varicosities establish more synapses [63]. The hypothesis of higher synaptic incidence for 5-HT varicosities that contain the VGLUT3 remains to be tested at the ultrastructural level.

## Supporting Information

Figure S1 **Confocal image of a BDA-injected axon travelling in the motor cortex.** Immunoreactivity for BDA, 5-HT and SERT are shown in red, green and blue, respectively. Note that all 5-HT-labeled neuronal elements are also immunoreactive for SERT. Arrow indicates a BDA axon varicosity immunoreactive for 5-HT and SERT. Lefts panels are from a 14 µm-thick Z-stack whereas the right panels are single plane representations. Scale bars = 5 µm.
(TIF)

Figure S2 **Schematic representation of 3 rostrocaudal transverse sections through the DRN showing the exact location of parent cell bodies.** The numbers refer to reconstructed neurons presented in Table S1.
(TIF)

Table S1 **Axonal branching patterns of reconstructed DRN neurons.**
(DOCX)
