# Supplementary figures and images for: Distribution of VGLUT3 in Highly Collateralized Axons from the Rat Dorsal Raphe Nucleus as Revealed by Single-Neuron Reconstructions
Source: PLoS One. 2014 Feb 4;9(2):e87709. doi: 10.1371/journal.pone.0087709 (PMC3913638; doi:10.1371/journal.pone.0087709)

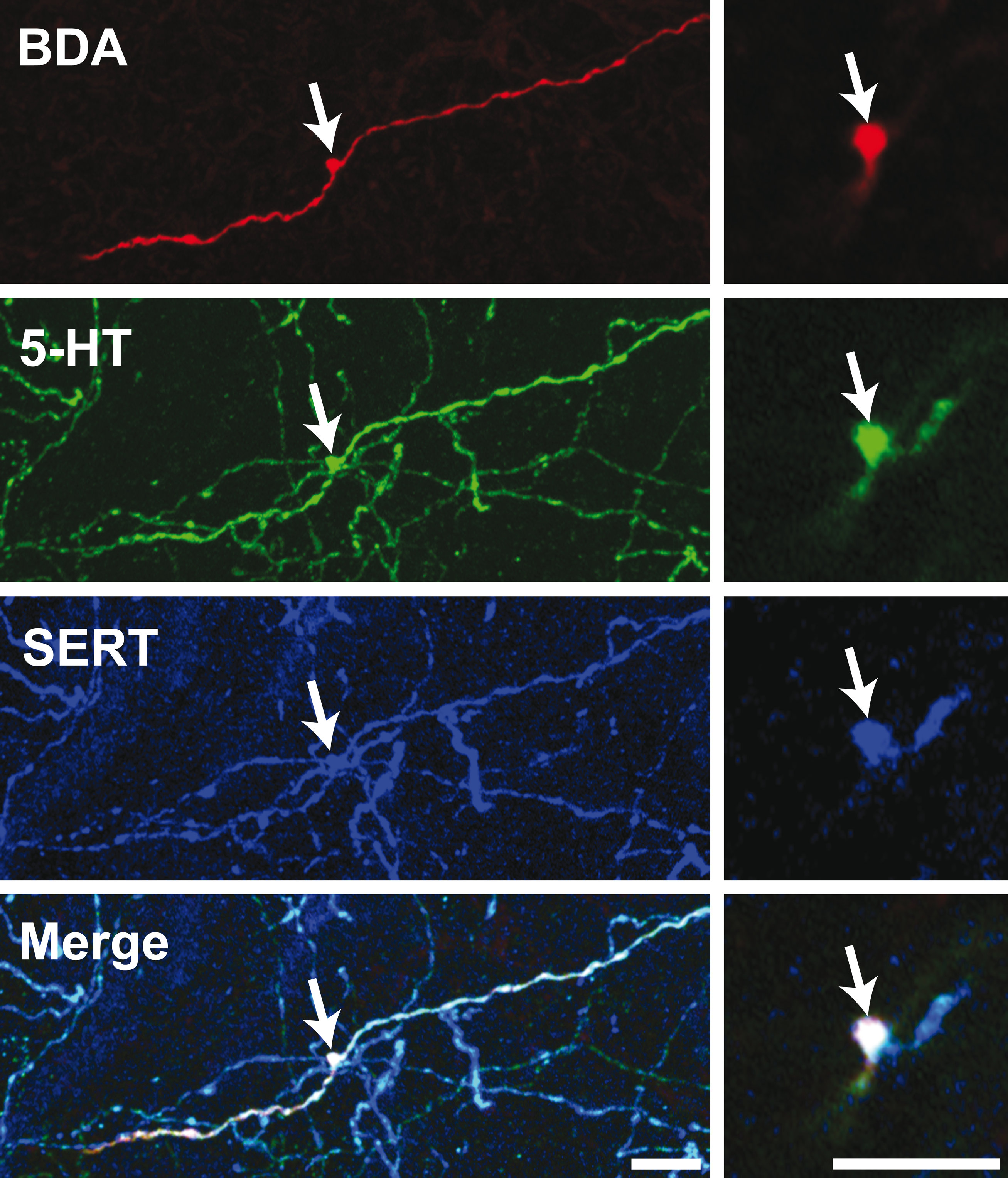

Supplement: Figure S1 — Confocal image of a BDA-injected axon travelling in the motor cortex. Immunoreactivity for BDA, 5-HT and SERT are shown in red, green and blue, respectively. Note that all 5-HT-labeled neuronal elements are also immunoreactive for SERT. Arrow indicates a BDA axon varicosity immunoreactive for 5-HT and SERT. Lefts panels are from a 14 µm-thick Z-stack whereas the right panels are single plane representations. Scale bars = 5 µm. (TIF) [file pone.0087709.s001.tif]

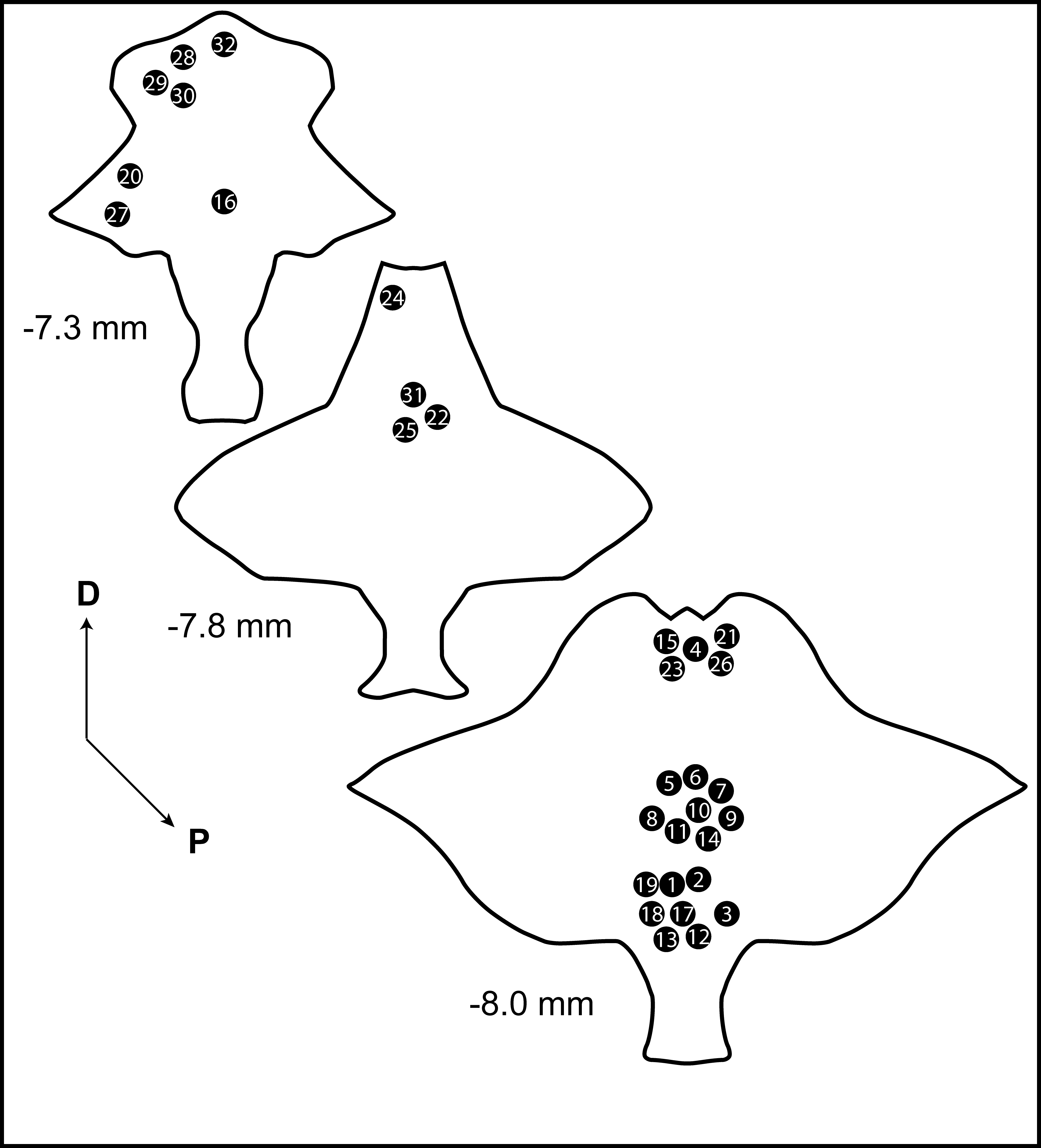

Supplement: Figure S2 — Schematic representation of 3 rostrocaudal transverse sections through the DRN showing the exact location of parent cell bodies. The numbers refer to reconstructed neurons presented in Table S1. (TIF) [file pone.0087709.s002.tif]
