# Supplementary material for: Distribution of VGLUT3 in Highly Collateralized Axons from the Rat Dorsal Raphe Nucleus as Revealed by Single-Neuron Reconstructions
Source: PLoS One. 2014 Feb 4;9(2):e87709. doi: 10.1371/journal.pone.0087709 (PMC3913638; doi:10.1371/journal.pone.0087709)
Supplement: Table S1 — Axonal branching patterns of reconstructed DRN neurons. (DOCX) [file pone.0087709.s003.docx]

*Table S1: Axonal branching patterns of reconstructed DRN neurons*

| ***#*** | ***Animal*** | ***Basal forebrain*** | ***Diencephalon and midbrain tegmentum*** | | ***Amygdala and septal area*** | | ***Cerebral cortex*** | | ***Hippocampus*** |
| --- | --- | --- | --- | --- | --- | --- | --- | --- | --- |
| *1* | A | STR (+++) |  | |  | |  | |  |
| *2* | A | STR (+++) |  | |  | |  | |  |
| *3 (Fig 2A)* | A | STR (+++) |  | |  | | PFC (++) | |  |
|  |  | Cl (+) |  |  |  |  |  |  |  |
|  |  | Tu (++) |  |  |  |  |  |  |  |
| *4 (Fig 2B)* | C | STR (++) | SN (+) | |  | | mCx (++) | |  |
|  |  | VP (+) |  |  |  |  |  |  |  |
|  |  | MCPO (+) |  |  |  |  |  |  |  |
| *5* | B | STR (+) | STN (+) | |  | | PFC (+) | |  |
|  |  | GP (+) |  |  |  |  |  |  |  |
| *6* | B | STR (++) | VM (+) | |  | |  | |  |
|  |  | GP (+) |  |  |  |  |  |  |  |
| *7* | B | STR (++) |  | |  | |  | |  |
|  |  | GP (++) |  |  |  |  |  |  |  |
| *8* | B | STR (+) | SN (+) | |  | | PFC (+) | |  |
|  |  | VP (+) |  |  |  |  |  |  |  |
| *9* | B | STR (++) | SN (++) | |  | | PFC (++) | |  |
|  |  | GP (+) |  |  |  |  |  |  |  |
| *10 (Fig 2D)* | B | Acb (+) | PC (++) | |  | |  | |  |
|  |  |  | PF (+) | |  |  |  |  |  |
|  |  |  | IHb (++) | |  |  |  |  |  |
|  |  |  | MB (+) | |  |  |  |  |  |
|  |  | MCPO (+) | VTA (+) | |  |  |  |  |  |
|  |  |  | LH (++) | |  |  |  |  |  |
|  |  |  | SUM (+) | |  |  |  |  |  |
| *11* | B |  | SUM (+) | |  | |  | |  |
|  |  |  | LH (+) | |  |  |  |  |  |
| *12 (Fig 2E)* | A |  | STN (++) | |  | |  | |  |
|  |  |  | SN (++) | |  |  |  |  |  |
| *13* | A |  | SN (++) | |  | |  | |  |
| *14* | B |  | MRN (+) | |  | |  | |  |
|  |  |  | VTA (+) | |  |  |  |  |  |
| *15* | C |  | SI (++) | |  | |  | |  |
|  |  |  | LH (+) | |  |  |  |  |  |
| *16* | E | VP (+) | VTA (++) | |  | |  | |  |
|  |  | Acb (+) |  |  |  |  |  |  |  |
| *17* | A |  | LH (+) | |  | |  | |  |
|  |  |  | PO (+) | |  |  |  |  |  |
| *18* | A | MCPO (+) | VTA (++) |  | |  | |  | |
|  |  |  | LH (++) |  |  |  |  |  |  |
| *19* | A |  | PF (+) |  | |  | |  | |
|  |  |  | PO (+) |  |  |  |  |  |  |
|  |  |  | LH (+) |  |  |  |  |  |  |
|  |  |  | ZI (+) |  |  |  |  |  |  |
| *20* | G |  | APTV (+) |  | |  | |  | |
|  |  |  | MGV (+) |  |  |  |  |  |  |
|  |  |  | ZI (++) |  |  |  |  |  |  |
| *21 (Fig 2C)* | C | Acb (+++) | LH (+) | BST (++) | |  | |  | |
| *22 (Fig 3A)* | D | Acb (++) | SUM (+) | Ce (++) | |  | |  | |
|  |  |  |  | BL (++) | |  |  |  |  |
| *23* | C |  | LH (+) | BST (+) | |  | |  | |
| *24* | E |  | LH (+) | LS (++) | |  | |  | |
| *25 (Fig 3B)* | D | VP (+++) | SUM (+) | BST (+) | |  | |  | |
|  |  |  |  | Ce (++) | |  |  |  |  |
|  |  |  |  | BL (+++) | |  |  |  |  |
| *26* | C | GP (+) |  | AA (+) | |  | |  | |
|  |  |  |  | Ce (++) | |  |  |  |  |
| *27 (Fig 3D)* | G |  |  |  | | PFC (++) | |  | |
| *28* | F |  |  |  | | PFC (++) | |  | |
| *29* | F |  |  | LS (+) | |  | | CA3 (+) | |
| *30* | F |  | SUM (+) |  | |  | | CA3 (+) | |
|  |  |  | LH (++) |  |  |  |  |  |  |
| *31* | D |  |  | LS (+) | |  | | DG (++) | |
| *32 (Fig 3C)* | F |  |  | LS (++) | | PFC (+++) | | CA1 (+++) | |
|  |  |  |  |  |  |  |  | CA2 (+++) | |
|  |  |  |  |  |  |  |  | CA3 (++) | |
|  |  |  |  |  |  |  |  | DG (++) | |

See figure S2 for location of parent cell bodies. AA, anterior amygdaloid area; Acb, accumbens nucleus; APTV, anterior pretectal nucleus; BL, basolateral amygdaloid nucleus; CA1, Ammon’s horn field 1; CA2, Ammon’s horn field 2; CA3, Ammon’s horn field 3; BST, bed nucleus of the stria terminalis; Ce, central amygdaloid nucleus; Cl, claustrum; DG, Dentate gyrus GP, Globus pallidus LH, lateral hypothalamic area; lHb, lateral habenula; LS, lateral septum; MB, mammillary body; MCPO, magnocellular preoptic nucleus; mCx, motor cortex; MGV, medial geniculate nucleus; MRN, median raphe nucleus; PC, paracentral thalamic nucleus; PF, parafascicular thalamic nucleus; PFC, prefrontal cortex; PO, preoptic area l; SI, substantia innominata; SN, substantia nigra; STN, subthalamic nucleus; STR, striatum; SUM, supramammillary nucleus; Tu, olfactory tubercle; VM, ventromedial nucleus of thalamus; VP, ventral pallidum; VTA, ventral tegmental area; ZI, zona incerta.
